# Social Confidence in Early Adulthood Among Young People With and Without a History of Language Impairment

**DOI:** 10.1044/2017_JSLHR-L-16-0256

**Published:** 2017-06-10

**Authors:** Kevin Durkin, Umar Toseeb, Nicola Botting, Andrew Pickles, Gina Conti-Ramsden

**Affiliations:** a School of Psychological Sciences and Health, University of Strathclyde, Glasgow, United Kingdom; b Department of Psychology, Manchester Metropolitan University, United Kingdom; c Language and Communication Science, City University, London, United Kingdom; d Department of Biostatistics, Institute of Psychiatry, King's College London, United Kingdom; e School of Health Sciences, The University of Manchester, United Kingdom

## Abstract

**Purpose:**

The purposes of this study were to test the predictions that lower self-esteem and higher shyness in individuals with a history of language impairment (LI) would continue from adolescence into early adulthood and that those with LI would have lower social self-efficacy in early adulthood.

**Method:**

Participants were young people with a history of LI and a comparison group of age-matched peers. Both groups were tested at ages 17 and 24 years. Participants completed measures of language ability, nonverbal IQ, shyness, global self-esteem, and (at age 24 years only) social self-efficacy.

**Results:**

Young adults with LI scored lower than age-matched peers on self-esteem, higher on shyness, and lower on social self-efficacy (medium to large effect sizes). In line with expectations, in the group with LI, language ability in adolescence predicted shyness in young adulthood, which, in turn, was negatively associated with self-esteem. There was also a direct association between language ability in adolescence and self-esteem in young adulthood.

**Conclusions:**

Young people with a history of LI are likely to be entering adulthood less socially confident than their peers. Interventions may be desirable for young adults with LI, and the present findings indicate social self-efficacy as a key area of social confidence that calls for practitioners' attention.

Young people growing up with language impairment (LI) face considerable burdens that extend beyond language difficulties themselves. Language is a primary tool for interacting with the world and for learning. As will be outlined in the following, difficulties in comprehending and/or producing language are associated in development with problems in other domains, such as behavior and social relations. This wide-ranging and protracted adversity could be expected to have negative consequences for the self-esteem and social confidence of individuals with LI as they reach adulthood, but, to date, only a small amount of evidence is available. We investigate the relationship between shyness and self-esteem scores collected in adolescence (17 years) and the shyness and self-esteem scores of the same individuals in young adulthood (24 years). We examine the relationships among language ability, shyness, and global self-esteem. In this research article, we also present (what is, to the authors' knowledge) the first comparison between young adults with and without LI on a measure of social self-efficacy (SSE).

## LI and Its Impact on Other Aspects of Development

Behavioral and emotional difficulties in children with LI are often more pronounced than population norms (Beitchman, Hood, Rochon, & Peterson, 1989; Botting & Conti-Ramsden, 2000; Cohen et al., 1998; Fujiki, Brinton, & Clarke, 2002; Petersen et al., 2013; Van Daal, Verhoeven, & Van Balkom, 2007). For many, these and related problems persist through middle childhood and into adolescence, with poorer mental health and poorer peer relations (Beitchman et al., 1996; Conti-Ramsden & Botting, 2004, 2008; Durkin & Conti-Ramsden, 2007; Fujiki, Spackman, Brinton, & Hall, 2004; Mok, Pickles, Durkin, & Conti-Ramsden, 2014; Yew & O'Kearney, 2013).

## LI and Self-Esteem in Childhood and Adolescence

Self-esteem is defined as the extent to which one values oneself (Cooley, 1902; Coopersmith, 1967). It can be measured for particular domains (e.g., educational self-esteem and physical self-esteem) and/or in terms of overall self-regard or global self-esteem (Rosenberg, 1965). In the present study, we will be concerned with global self-esteem, as measured by the Rosenberg Self-Esteem Scale (RSES; Rosenberg, 1965). This instrument gauges how the individual perceives her or his general worth, possession of positive qualities, and whether or not she or he takes a positive attitude toward the self. Global self-esteem is important because it bears on individuals' personal goals, beliefs about their worth, and expectations about their future. In the general population, those with high self-esteem tend to achieve more favorable outcomes in many aspects of life, whereas those with low self-esteem tend to fare less well (Orth, Robins, & Widaman, 2012). For example, in a large-scale prospective study, Trzesniewski et al. (2006) found that adolescents with low global self-esteem grew up to have poorer mental health and poorer economic outcomes in their mid-20s.

It seems very plausible that LI and its associated difficulties would tend to depress self-esteem in young people. It is potentially undermining to find oneself less able than others to handle everyday communication, to be on the periphery of peer groups, or to be performing poorly in the classroom. The evidence available for self-esteem, however, is mixed. For example, some studies of elementary school–age children (6- to 9-year-olds) have reported null results when comparing self-esteem in those with and without LI (Jerome, Fujiki, Brinton, & James, 2002; Lindsay & Dockrell, 2000; Lindsay, Dockrell, Letchford, & Mackie, 2002). It may be that children in this age range are not yet fully aware of the ways in which they are different to others (Jerome et al., 2002). Older children and adolescents in the same studies, on the other hand, did have lower self-esteem, particularly in relation to social and academic domains. Furthermore, in a study of adolescents aged 16 to 17 years completing the RSES (Rosenberg, 1965), Wadman, Durkin, and Conti-Ramsden (2008) found that those with a history of LI had significantly lower scores than did peers with typical development, with a medium effect size. Note that the overall means of both groups approximated to the mean RSES scores reported in many studies of typical populations (Schmitt & Allik, 2005). Even so, the LI mean was lower than that of the age-matched peer (AMP) group, and some 48% of participants with LI had self-esteem scores below the norm for their age group, while this was the case for only 11% of the AMP group. Lindsay, Dockrell, and Palikara (2010), in a longitudinal study, monitored change in self-esteem in a sample of young people with LI over a similar age range to Wadman et al. (2008). They found improvements from ages 16 to 17 years for several domains of self-esteem (perceptions of scholastic competence, job competence, global self-worth, physical appearance, athletic competence) but not in interpersonal domains (social acceptance, close friendships, and romantic relationships).

In short, the evidence to date suggests that a negative relationship between LI and self-esteem is not reliable in middle childhood but is apparent by midadolescence. Some evidence indicates that increases in self-esteem may occur in those with LI during midadolescence (approximately ages 16 to 17 years), but self-esteem relating to social domains may be less likely to increase. Where discrepancies between those with and without LI have been reported, they are not necessarily enormous, but those with LI tend to be disadvantaged.

## LI and Shyness in Childhood and Adolescence

Shyness is the feeling of tension, discomfort, and inhibition in the presence of others (Cheek & Buss, 1981). For example, individuals who score high on shyness tend to perceive themselves as socially awkward, are uncomfortable in social contexts, and often find it difficult to adapt to new social settings. In the general population, individuals differ in the extent to which they experience shyness, and it is generally regarded as an enduring personality characteristic. Nevertheless, it can be subject to some within-individual variability, too (across situations and/or over time; Ash, Rice, & Redmond, 2014; Liu et al., in press). For example, children learning English as a second language score higher on measures of shyness when in English-speaking contexts than when in contexts in which they are able to use their native language (Ash et al., 2014). High levels of shyness are associated with negative self-perceptions, internalizing symptoms, limited social participation, problems or delays in relationship formation, peer rejection or victimization, and poorer career development (Coplan, Closson, & Arbeau, 2007; Coplan & Rubin, 2010; Phillips & Bruch, 1988; Rowsell & Coplan, 2013). Shyness is negatively correlated with self-esteem (Crozier, 1995; Lawrence & Bennett, 1992; Wadman et al., 2008).

Considerable evidence exists to establish that there is a negative association in childhood between language ability and shyness (Coplan & Evans, 2009). The causal directions underpinning the association are not fully understood (Coplan & Evans, 2009; Smith Watts et al., 2014). One possibility is that having LI makes interacting with others uncomfortable and that some children respond to this by avoiding or withdrawing from social contexts. On the other hand, it is possible that those who do not have the motivation or desire to participate socially will find less opportunity to practice or extend their language abilities. Another possibility is that deficits in both language and social confidence reflect some common underlying cause, which could be genetic or environmental. Smith Watts et al. (2014) tested several competing hypotheses in a longitudinal study of a large sample of toddlers. The authors found some evidence consistent with the proposition that lower expressive language ability led to higher growth of behavioral inhibition and some evidence consistent with the proposition that shy children do know more about language than they demonstrate but are reluctant to express it. The findings of Smith Watts et al. (2014), however, concern relations among variables in toddlers, none of whom were identified as having LI. Causal relations may change with development, and causal relations may be different in those with and without LI. For example, although it is plausible that shyness could inhibit language use in some contexts, it seems less plausible that it could bring about the pervasive linguistic difficulties found in those with LI, and no theory has been advanced to propose that LI is caused by shyness. These considerations prompt a need for evidence on the patterns of relationship in those with LI, including relationships later in development.

Numerous investigations concur that individuals with LI, from early in childhood, tend to be more reticent (wary or even fearful of joining social groups), less likely to initiate interactions, and poorer at maintaining conversations than are their typically developing peers (Conti-Ramsden & Botting, 2004; Fujiki & Brinton, 2015; Fujiki, Brinton, Morgan, & Hart, 1999; Fujiki et al., 2004; Hart, Fujiki, Brinton, & Hart, 2004; Maggio et al., 2013). In adolescence, individuals with LI tend to be significantly shyer than their peers (Wadman et al., 2008). Wadman et al. argued (after Redmond & Rice, 1998) that this can be explained as the consequence of an adaptive process. Language difficulties make it challenging to interact with others, so the young person seeks to reduce the discomfort by restricting, or even withdrawing from, social interactions. It follows that if there is a relationship between LI and self-esteem, it should be at least partially dependent on the extent to which the individual manifests shyness. Wadman et al. (2008) tested this possibility in their adolescent sample and found evidence that a relationship between language ability and global self-esteem (RSES) was, as predicted, partially mediated by shyness.

In short, from early childhood, individuals with LI tend to be reticent and less socially engaged. In adolescence, they are significantly shyer than peers without LI. Through development, LI is associated with, leads to, or exacerbates shyness, and shyness contributes to lower self-esteem.

## Social Confidence in Individuals with LI in Early Adulthood

What is the legacy of this developmental history as young people with LI reach adulthood? How confidently do individuals with LI approach the tasks of entering society?

Relevant research is somewhat scarce, but there is a growing body of evidence that the nonlinguistic difficulties associated with LI in childhood continue through adolescence and into adulthood. For example, young adults with a history of early LI have significantly higher rates of mental health problems (Beitchman et al., 2001; Conti-Ramsden & Botting, 2008; Law, Rush, Schoon, & Parsons, 2009; Schoon, Parsons, Rush, & Law, 2010). Difficulties in social adjustment and friendship maintenance are often reported in young adults with LI (Clegg, Hollis, Mawhood, & Rutter, 2005; Törnqvist, Thulin, Segnestam, & Horowitz, 2009; Whitehouse, Watt, Line, & Bishop, 2009). Voci, Beitchman, Brownlie, and Wilson (2006) found that the incidence of social phobia (fear of interacting with others, speaking to small or large groups, and being observed) was more than twice as high in 19-year-olds with LI (who had been identified first at age 5 years) than in peers with typical language skills. Indeed, the authors observe that the rate of social phobia in the group with LI was one of the highest reported in the epidemiological literature.

This developmental context leads to the possibility of a lower sense of social effectiveness and confidence than might be expected in a sample of young adults without LI. The disadvantages that affect self-esteem during adolescence could also be expected to be sustained into adult life. Individuals who tended to be reticent or socially marginal in childhood and adolescence may be likely to remain shyer than average into adult life.

A construct that is closely related to, but distinct from, shyness is SSE, which is “an individual's confidence in her/his ability to engage in the social interactional tasks necessary to initiate and maintain interpersonal relationships” (Smith & Betz, 2000, p. 286). For example, a person with high SSE would regard himself or herself as able to initiate a conversation with someone unfamiliar, able to handle unfamiliar social situations, and knowing how to relate to others effectively. Although shyness is a personality trait, SSE is conceived of as a product of social-cognitive activity: it emerges from individuals' reflections on their prior experiences, and it guides their expectations about how they are likely to perform in the future (Bandura, 1997). Thus, via self-referent cognitions about their social competence, individuals contribute causally to their own personal development (Caprara, Steca, Cervone, & Artistico, 2003).

SSE, then, has the potential to be a revealing measure of how confidently young people with LI feel they can manage a host of social demands that are encountered in everyday life and workplaces in adulthood. The SSE scale developed by Smith and Betz (2000) asks participants to indicate, in each case on a scale anchored from 1 = *no confidence at all* to 5 = *complete confidence,* their abilities for items such as “Express your opinion to a group of people discussing a subject that is of interest to you”; “Work on a school, work, community or other project with people you don't know”; “Put yourself in a new and different social situation”; “Be involved in group activities”; “Ask someone for help when you need it”; and “Call someone you've met and would like to know better” (35 items in all). SSE is associated negatively with shyness and positively with global self-esteem (Caprara & Steca, 2005; Smith & Betz, 2000; Watson & Nesdale, 2012).

Although there are strong reasons to expect a developmentally based disadvantage to young adults with LI regarding social confidence (that is, self-esteem, shyness, and SSE), note that, theoretically, there are alternative possibilities. For example, young adulthood is a major new life phase that offers greater status and freedom and many opportunities and rewards; for most young people, this is a time of gains in autonomy and relative optimism about their personal future (Arnett, 2004). Consistent with this, cross-sectional and longitudinal studies (in the general population) show gradually increasing self-esteem scores from around 18 years through early adulthood (Orth et al., 2012; Robins & Trzesniewski, 2005). We lack data on whether young adults with LI show a similar trajectory.

Although there are continuities in relative shyness through childhood and into adulthood (Dennissen et al., 2008; Schwartz, Snidman, & Kagan, 1999), there are some indications that shyness declines slightly in the general population during early adulthood (Dennissen et al., 2008; Grühn et al., 2010). It has been argued that this may reflect experience-based growth in social competence (Grühn et al., 2010). For example, by this stage of life, individuals may have developed strategies for how to respond to unfamiliar people (Dennissen et al., 2008). For young adults with LI, however, there is less evidence on within-individual continuities from earlier in development and on whether there is an overall reduction in shyness in early adulthood.

## The Present Study

Global self-esteem, shyness, and SSE are all important indicators of how adequately a young person is equipped psychologically to meet confidently the myriad challenges and opportunities of adult life. In each case, there are reasons to suppose that individuals who have grown up with LI will be at a disadvantage compared with typical peers, although alternative outcomes or null findings are conceivable. To date, relatively little direct evidence is available. The first purpose of the present study was to provide such evidence, on the basis of a sample of individuals with LI who had been followed from childhood to early adulthood. We predicted that, at age 24 years, these participants would score lower on measures of global self-esteem, shyness, and SSE than would AMPs without LI. We also conducted a correlational analysis of the relationships among global self-esteem, shyness, and SSE in both groups of participants. These are known to be interrelated constructs in the typical population, but it is not known if the pattern of relationship is identical in those with LI and, importantly, it is not known if language ability is significantly associated with these constructs.

The second purpose of the study focused on shyness. We examined the relationship between shyness scores of young people with LI in adolescence (17 years) and the shyness scores of the same individuals in young adulthood (24 years). Again, although shyness is a relatively enduring trait, it can vary over time, and some individuals become less shy as they gain experience and confidence in handling social situations (Asendorpf, 2000). In a similar way, we examined correlations between self-esteem scores obtained at the same two time points. Self-esteem is also amenable to variation over time (Eccles et al., 1989; Lindsay et al., 2010; Robins & Trzesniewski, 2005). For each variable, little previous evidence is available speaking to continuity or change in those with LI from adolescence to early adulthood.

The third purpose of the study was to test causal models of the relationships among language ability, shyness, and global self-esteem. In particular, we expected that language ability, measured at ages 17 and 24 years, would predict self-esteem at 24 years of age and that this relationship would be, at least partially, mediated by shyness among participants with LI. We did not expect to find such a mediation effect among peers without LI. There was no evidence, for example, of such a relationship in typically developing individuals at age 17 years (Wadman et al., 2008). There is no reason to assume that variation within the normal range of language ability should itself provoke the emergence of shyness. Thus, although shyness is a normally distributed trait among the general population and could well affect self-esteem, this relationship should, among those without LI, be independent of language ability.

## Method

### Ethics

The study reported here received ethical approval from the University of Manchester, United Kingdom. All participants provided informed written consent.

### Participants

#### Participants With a History of LI

In the current investigation, there were 90 young adults with a history of LI at 17 years and 84 at 24 years, who were originally part of a wider study, the Manchester Language Study. There were 62 participants who provided data at both time points. The sample at 17 years, 37% of the original cohort, consisted of 62 (69%) men and 28 (31%) women. The sample at 24 years, 35% of the original cohort, consisted of 56 (67%) men and 28 (33%) women. The initial cohort of 242 children were recruited from 118 language units across England and represented a random sample of 50% of all 7-year-olds attending language units for at least half of the school week. Language units are specialized classes for children who have been identified with LI (i.e., primary language difficulties). Language unit placements are offered to children who would find it difficult to cope in mainstream education, even with support. These children are deemed to need a structured small group setting with intensive language input that usually involves both teachers and speech-language therapists.

#### AMPs

The comparison group consisted of 91 AMPs aged 17 years and 88 aged 24 years who had no history of special educational needs or speech and language therapy provision. There were 57 AMPs who took part at both time points. At the age of 17 years, there were 54 (59%) men and 37 (41%) women. At the age 24 years, there were 49 (56%) men and 39 (44%) women. Sixty-six of these young adults were recruited at age 16 years and 22 young adults were recruited age 24 years. Participants at 16 years of age came from the same schools as the participants with a history of LI, as well as additional targeted schools. These participants were sampled from selected demographic areas to ensure AMPs came from different backgrounds and wide geographical areas, similar to participants with a history of LI. The 22 young adults were recruited to match the original sample in terms of age and socioeconomic status, as measured by personal income. Thus, the LI and the AMP groups did not differ on personal income at age 24 years, χ^2^(5, *N* = 131) = 7.38, *p* = .194.

### Materials

#### Language

The Clinical Evaluation of Language Fundamentals–Fourth Edition UK (CELF-4 UK; Semel, Wiig, & Secord, 2006) was used to assess language ability. A core language index was calculated by using both receptive and expressive language subscales following the procedure specified in the CELF manual. Given the dearth of standardized language tests in adulthood and for continuity, the CELF-4 UK was deemed the best fit assessment for our cohort at age 24 years (neither group reached ceiling levels on this assessment, which is normed up to age 21;11 years). The CELF-4 UK has good reliability in young adulthood (i.e., reliability for the word classes subtest is reported to be .88 and for the formulated sentences subtest is .82). Clinical validation studies of the CELF-4 UK reported in the manual indicate that the test is sensitive to LI in children, adolescents, and young adults.

#### Nonverbal IQ

The Wechsler Abbreviated Scale of Intelligence (Wechsler, 1999) Performance subscale was administered as a measure of nonverbal IQ. Standard scores were calculated.

#### Shyness

The revised Cheek and Buss Shyness Scale (Cheek, 1983) was used to measure shyness. Versions of this scale are used in most social psychological and personality research into shyness. The adapted version of the scale, also previously used by Stritzke, Nguyen, and Durkin (2004) and Wadman et al. (2008), consisted of 12 items to which participants responded by using a 5-point scale (e.g., 1 = *very uncharacteristic,* 2 = *characteristic,* 3 = *neither characteristic nor uncharacteristic,* 4 = *uncharacteristic,* 5 = *very characteristic*). Items included “I am often uncomfortable at parties and other social functions” and “I am socially awkward” and also contained items that were reversed scored, such as “It does not take me long to overcome my shyness in new situations.” Scores were summed and a higher score indicated higher levels of shyness. Satisfactory convergent and discriminant validity have been established for the revised Cheek and Buss scale (Hopko, Stowell, Jones, Armento, & Cheek, 2005). The internal reliabilities in the present administration were very high (Cronbach's α: LI = .87 and AMP = .89).

#### Global Self-Esteem

The RSES (Rosenberg, 1965) was used to measure global self-esteem. This is the most widely used instrument in research assessing self-esteem in adolescents and adults. The scale consists of 10 items each requiring the participants to respond on a 4-point scale (1 = *strongly* *agree,* 2 = *agree,* 3 = *disagree,* or 4 = *strongly disagree*). Items included “I feel I have a number of good qualities” and “At times I think I am no good at all,” as well as reversed scored items, such as “I take a positive attitude toward myself.” Higher summed scores indicated higher self-esteem. Satisfactory convergent, discriminant, and predictive validity for the RSES has been well-documented (Blascovich & Tomaka, 1991; Rosenberg, 1965). In the present administration, the internal reliabilities were very high for each group (Cronbach's α: LI = .88 and AMP = .89).

#### SSE

The Perceived Social Self-Efficacy Scale (Smith & Betz, 2000) was used to measure self-efficacy. The scale consists of 25 items scored on a 5-point Likert-type scale (1 = *no confidence at all,* 5 = *complete confidence*). Items include the following: “Start a conversation with someone you don't know very well”; “Put yourself in a new and different social situation”; and “Be involved in group activities.” Given the nature of SSE and the confidence rating scoring system, the Perceived Social Self-Efficacy Scale does not include any negatively worded items that would require reverse scoring. Higher summed scores indicated higher SSE. This measure was administered at age 24 years only. Good evidence of convergent validity and high reliabilities have been reported for this instrument (Smith & Betz, 2000). In the present administration, the internal reliabilities were very high for each group (Cronbach's α: LI = .96 and AMP = .96).

#### Procedure

The participants were interviewed face-to-face at their educational institution or home at age 17 years and at their home at age 24 years, as part of a wider battery of tests. Interviews took place in a quiet room, wherever possible, with only the participant and a trained researcher present. Basic demographic information was collected, and then the standardized assessments were administered in the manner specified by the test manuals. For the interview, the items were read aloud to the participants, and the participants were given additional clarification, where needed, although this occurred rarely. Particular care was taken to ensure the participants understood the interview items. The response options were carefully explained, and both the items and response options were also presented visually. Participants could respond verbally or by pointing to the response options presented visually.

### Statistical Analysis

All statistical analyses were conducted in Stata/SE Version 13.1 (StataCorp, 2013). A two-tailed significance level of *p* = .05 was used, unless otherwise specified. Independent *t* tests were used to compare group differences between groups in language, nonverbal IQ, shyness, global self-esteem, and SSE. Following this, within-individual continuity and change between ages 17 and 24 years was examined by using pairwise correlations for all measures, except SSE, as this was only collected at 24 years of age. Relationships across variables were also investigated. In particular, the relationship of language with shyness, self-esteem, and SSE was examined in each of the groups, as it is not known if the pattern of associations is similar in young people with and without LI.

A further aim of the study was to examine the relationships among language ability, shyness, and self-esteem; thus, a mediation analysis was undertaken. Mediation analysis affords better understanding (Baron & Kenny, 1986) of underlying mechanisms or processes by which one variable (e.g., language) influences another variable (e.g., self-esteem) through a mediator variable (e.g., shyness). Mediation conceptually means causation. However, mediation analysis must be carried out and interpreted with caution, because measurement of mediation, statistically, is, in essence, a set of correlations. Therefore, it is possible that some other variable, strongly correlated with the key variable of interest but not considered in the analysis, may, in fact, be the causal factor. For example, in the case of language abilities, variables collinear with language abilities, such as school success, socioeconomic status, nonverbal, or other skills, may be, in fact, the causal agents.

Nonetheless, there are stronger reasons to suppose that LI influences self-esteem than that self-esteem gives rise to LI. Theoretical arguments and empirical data support the examination of shyness as a potential mediating variable in the relationship between language and self-esteem in young people with LI (Wadman et al., 2008). Within this context, a specific mediation analysis, following Hayes (2009), was conducted for the group with LI to investigate the mediating effect of shyness (age 24) on the relationship between language (age 17 years) and global self-esteem (age 24 years). Regression equations were estimated simultaneously by using the Structural Equation Modeling (SEM) command in Stata/SE. Missing data were treated as such, and only available data were analyzed.

## Results

### Group Comparisons: Psycholinguistic Profiles, Shyness, Self-Esteem, and Self-Efficacy

The mean standard scores, standard deviations, and LI versus AMP comparisons on language and nonverbal measures are presented in Table 1. The AMP participants had mean receptive, expressive, and core language scores within the expected range. At both time points, the participants with a history of LI had significantly lower receptive, expressive, and core language scores; mean scores fell below 1 standard deviation below the mean (< 85). Both groups of young adults had mean nonverbal IQ within the expected range at both time points. Note, nonetheless, that the young adults with a history of LI had significantly lower nonverbal IQ scores than their peers, as is often found in research with this population (Leonard, 2014).

**Table 1.** Group differences for variables of interest at ages 17 and 24 years.

Variable	Age 17 years				Age 24 years			
	LI *M* (*SD*)	AMP *M* (*SD*)	*t* statistic	Effect size *d*	LI *M* (*SD*)	AMP *M* (*SD*)	*t* statistic	Effect size *d*
Receptive language	75.8 (17.9)	99.9 (12.1)	*t*(179) = −10.6***	−1.6	83.5 (18.6)	106.2 (8.9)	*t*(168) = −10.2***	−1.6
Expressive language	73.3 (16.8)	100.2 (13.4)	*t*(176) = −11.9***	−1.8	81.6 (18.9)	105.6 (12.1)	*t*(167) = −9.9***	−1.5
Core language	68.9 (18.4)	102.6 (13.7)	*t*(178) = −13.9***	−2.1	69.3 (20.7)	100.0 (13.9)	*t*(167) = −11.3***	−1.7
Nonverbal IQ	93.4 (16.5)	106.4 (10.9)	*t*(179) = −6.2***	−0.9	98.8 (15.8)	111.9 (10.3)	*t*(167) = −6.4***	−1.0
Shyness	34.8 (8.1)	27.5 (8.1)	*t*(179) = 6.0***	0.9	35.3 (9.4)	25.9 (8.3)	*t*(165) = 6.8***	−1.1
Global self-esteem	30.1 (4.0)	32.1 (3.6)	*t*(179) = −3.5***	−0.5	30.0 (4.8)	32.8 (4.4)	*t*(165) = −3.9***	−0.6
Social self-efficacy	—	—	—	—	77.8 (19.9)	92.7 (17.8)	*t*(164) = −5.1***	−0.8

*Note.* LI = participants with a history of language impairment; AMP = age-matched peer. Correlations between 17- and 24-year measures of core language = .9; nonverbal IQ = .9; shyness = .6; and self-esteem = .6. Em dashes indicate data not obtained at this age level.

*** *p* < .001.

Descriptive statistics for between-groups comparisons of scores on shyness, global self-esteem, and SSE are presented in Table 1. As can be seen, there were strong differences in the predicted direction, with the group with LI scoring significantly lower on global self-esteem and SSE but higher on shyness.

### Longitudinal Associations Among Language, Shyness, Self-Esteem, and SSE

A correlation matrix for the key measures in young adulthood is shown in Table 2. There were a number of similarities across groups. For both groups, better language at 17 years was associated with better language at 24 years. Higher levels of shyness at age 17 years were associated with higher levels of shyness at age 24 years, with lower self-esteem (17 and 24 years), and with lower SSE (measured only at 24 years). Higher self-esteem at 17 years was associated with higher self-esteem at 24 years. For the AMPs only, higher self-esteem at age 17 years was also associated with higher SSE at age 24 years.

**Table 2.** Zero-order correlations between variables of interest.

Variable and age	1	2	3	4	5	6	7
1. Core language 17 years	1						
2. Core language 24 years		1					
Overall	0.9***						
LI	0.9***						
AMP	0.8***						
3. Shyness 17 years			1				
Overall	0.4***	-0.3***					
LI	−0.2*	−0.3*					
AMP	0.0^NS^	0.1^NS^					
4. Shyness 24 years				1			
Overall	0.4***	−0.4***	0.6***				
LI	−0.3*	−0.3**	0.5***				
AMP	−0.2^NS^	0.1^NS^	0.6***				
5. Global self-esteem 17 years					1		
Overall	0.3***	0.2*	−0.6***	−0.3***			
LI	0.3*	0.2^NS^	−0.6***	−0.2^NS^			
AMP	0.1^NS^	−0.1^NS^	−0.6***	−0.3*			
6. Global self-esteem 24 years						1	
Overall	0.4***	0.3***	−0.4***	−0.6***	0.4***		
LI	0.4**	0.2^NS^	−0.3*	−0.5***	0.3*		
AMP	0.3^NS^	0.0^NS^	−0.3*	−0.6***	0.4**		
7. Social self-efficacy 24 years							1
Overall	0.4***	0.3***	−0.6***	0.8***	0.3***	0.6***	
LI	0.3^NSS^	0.2*	−0.5***	−0.7***	0.2^NS^	0.5***	
AMP	0.1^N^	−0.1^NS^	−0.6***	−0.8***	0.4**	0.6***	

*Note.* Overall = LI and AMP groups combined; LI = participants with a history of language impairment; AMP = age-matched peer.

^NS^Not significant.

* *p* < .05.

** *p* < .01.

*** *p* < .001.

Relationships between language and the other variables revealed differences across the groups. For the young people with LI, better language at 17 and 24 years was associated with lower levels of shyness (age 17 and 24 years) and with higher self-esteem (age 17 and 24 years). Better language at 24 years was also associated with higher SSE at 24 years. In contrast, for the AMPs, there were no significant associations between language at 17 years, nor language at 24 years, and shyness, self-esteem, and SSE at any of the time points examined.

### Shyness in Young Adulthood as a Mediator of the Relationship Between Language and Self-Esteem

We tested the prediction that shyness at 24 years would mediate the relationship between language ability at age 17 years and self-esteem at age 24 years by using mediation analysis. For the participants with LI, the prerequisite condition for mediation analysis was met—that is, language ability at 17 years was directly and significantly associated with self-esteem at 24 years. For the AMPs, there was no association between language and self-esteem, so issues of mediation did not arise in this group.

**Figure 1.**
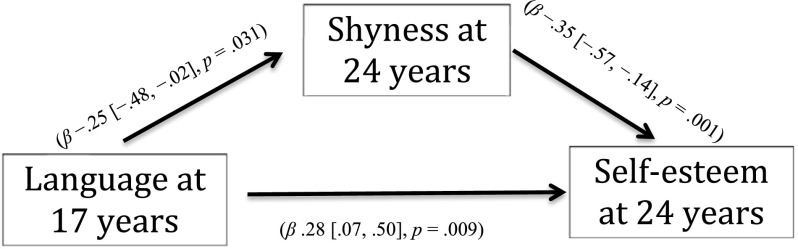
Shyness as a mediator of the relationship between language and self-esteem.

As shown in Figure 1, for the young people with LI, better language in adolescence was associated with being less shy in young adulthood, which was, in turn, associated with having higher self-esteem in young adulthood. Better language was also still directly associated with higher self-esteem. The proportion of the total effect that was mediated was .24. That is, 24% of the association between language and self-esteem was explained by shyness. The ratio of the indirect to direct effect is .3 and the total effect was approximately 1.3 times the indirect effect.

The association between language at 24 years and self-esteem at 24 years was weaker overall and was not significant in either group examined separately. Hence, there was no basis for testing the contemporaneous mediation model.

## Discussion

By the time they arrive at early adulthood, young people with LI are likely to have encountered many difficulties and unfavorable experiences in terms of their personal adjustment and relations to others. The present study tested aspects of the outcomes for individuals' confidence that they were able to effectively meet the challenges of the social world. As anticipated, 24-year-olds with LI scored lower on self-esteem (with a moderate effect size) and higher on shyness (with large effect size) than did AMPs without LI. This investigation reports for the first time significantly lower SSE in young adults with LI as compared with their peers. The size of the effect observed was large. Growing up with a history of LI is associated with disadvantage in these measures of social confidence, extending at least into early adulthood. This is important new evidence that the sequelae of LIs are sustained and potentially handicapping well beyond childhood and, for a number of individuals with LI, well beyond the age range during which they are likely to receive any language therapy or support.

Furthermore, each of shyness, self-esteem, and SSE are known to predict life outcomes in subsequent adulthood. Shyer individuals arrive at personal (e.g., finding a partner and becoming a parent) and career decisions (e.g., finding a profession and seeking promotion) later than do nonshy people (Caspi, Elder, & Bem, 1988). Those with lower self-esteem are at risk of poorer mental health and poorer economic outcomes (Trzesniewski et al., 2006). SSE, a social cognitive construction about one's competence to handle social tasks (Bandura, 1997; Caprara et al., 2003), predicts social competence, emotional well-being, and career progress (Rice, Cunningham, & Young, 1997; Smith & Betz, 2000). Thus, by their early 20s, the age of the present participants, a number of individuals with LI have disadvantageous developmental histories and are at greater risk of pursuing less rewarding futures.

The findings for self-esteem and shyness support findings in this same sample at age 17 years (Wadman et al., 2008). SSE was not measured at the earlier age, and the present findings make clear that there are substantial group differences in this respect, too, at age 24 years. We examined the relationships between shyness scores at the two age points and between self-esteem scores at the two age points. Each relationship was positive and moderate.

The relative standing of those with LI compared with those without LI remains constant but there are individual differences in the extent to which scores vary over time. Some fluctuation in shyness and self-esteem over time is consistent with theoretical arguments that these characteristics reflect adaptive processes (Asendorpf, 2000; Ash et al., 2014; Redmond & Rice, 1998; Wadman et al., 2008); changes in circumstances, skills, and/or a range of other experiences may have contributed to within-participant changes in shyness. Further research would be valuable in identifying which factors in the lives of young people with LI during the years of the early adult transition lead to variability and change in shyness scores. Nevertheless, the overriding finding is that, at age 24 years, the group with LI remained significantly shyer and less self-assured than their peers.

The findings also provide novel information on the potential causal relations among language ability, shyness, and global self-esteem. We expected that among participants with LI, language ability in adolescence would predict self-esteem and that this relationship would be at least partially mediated by shyness (as found in adolescence; Wadman et al., 2008). In line with expectations, we did find that in the group with LI, language ability in adolescence predicted shyness in young adulthood, which, in turn, was negatively associated with self-esteem. There was also a direct association between language ability in adolescence and self-esteem in young adulthood. This pattern was not observed in AMPs. This result suggests that having average (or above) language skills in midadolescence may be a variable that mitigates the risk of developing lower social confidence in the transition to young adulthood.

Note that the effects indicated in the correlations and regression analysis were modest, reflecting the heterogeneity of individuals with LI (Conti-Ramsden & Durkin, 2016) and that likelihood that multiple variables bear on the social confidence of young people with LI (see the following). Sample size restrictions meant that we could not enter every possible covariate. However, we did examine the consequences of including nonverbal IQ at 17 years as a mediator of the relationship between core language at 17 years and self-esteem at 24 years; the mediation effect for shyness was still obtained, albeit slightly attenuated. Future research with larger samples available could examine a wider range of possible covariates.

Note that the corresponding contemporaneous (age 24 years) pattern of relationships among language ability, shyness, and self-esteem was not as evident as that found between language at age 17 years and shyness and self-esteem at age 24 years. The dynamics of these relationships appear to change in the transition to young adulthood. This is interpretable from a developmental perspective. It suggests that having LI around midadolescence puts the young person at risk of disadvantage in terms of self- and social confidence over the next few years. This is analogous to other well-established developmental effects. For example, children who suffer parental neglect as toddlers or individuals who enter puberty markedly early or late are likely to continue to experience negative sequelae well into adulthood (Cicchetti & Toth, 2005; Cicchetti, Toth, Nilsen, & Manly, 2016; Mendle & Ferrero, 2012). The parental abuse or differences in sexual maturation itself are no longer in place by adulthood (e.g., there is no longer a contemporaneous correlation between secondary sexual characteristics and adjustment), but the psychological consequences of the earlier circumstances continue to have ramifications. Differences in language ability are still in place in early adulthood (see Table 1), but the evidence obtained here indicates that the causal links are distal (can be traced to the adolescent period) rather than proximate (cf. Belsky, Steinberg, & Draper, 1991).

Note that many other factors that interweave with language abilities are likely to bear on the development of social confidence in childhood, adolescence, and young adulthood in people with LI. For example, we know that having LI is associated with a range of social, cognitive, emotional, and behavioral difficulties from early in childhood (Andrés-Roqueta, Adrian, Clemente, & Villanueva, 2016; Durkin & Conti-Ramsden, 2010; Nilsson & López, 2016; Redmond & Rice, 1998; Schoon et al., 2010; Timler, Olswang, & Coggins, 2005). These characteristics themselves are likely, in turn, to be associated with risks and disadvantages, and relationships can be reciprocal: A child who is behaviorally challenging and emotionally volatile may find it difficult to make friends and thus have less opportunities to develop social skills. In a similar way, children with LI are at greater risk of having co-occurring conditions, such as attention-deficit/hyperactivity disorder, above-average autism spectrum disorder symptomatology, or motor disorders (Conti-Ramsden, Simkin, & Botting, 2006; Hill, 2001; Redmond, 2016), and when present, these characteristics, too, have implications for many other aspects of the individuals' lives (Durkin, Conti-Ramsden, & Simkin, 2012; Redmond, 2016). Children with LI are at greater risk of victimization (Brownlie, Jabbar, Beitchman, Vida, & Atkinson, 2007; Conti-Ramsden & Botting, 2004; Knox & Conti-Ramsden, 2007; Lindsay, Dockrell, & Mackie, 2008; Redmond, 2011). Experiencing peer victimization is associated with depressive symptoms, lower self-esteem, social phobia, and social exclusion (Blood & Blood, 2004; Hawker & Boulton, 2000; Hunter, Durkin, Heim, Howe, & Bergin, 2010; Liu et al., in press). As observed by Johnson, Beitchman, and Brownlie (2010), “a history of language disorder does not, in and of itself, predetermine outcomes for individuals” (p. 60). However, having LI places the child at risk of negative sequelae, and/or LI often co-occurs with other symptoms, disorder(s), or experiences that are associated with additional problems (Law, Reilly, & Snow, 2013).

### Clinical Implications

We have identified the patterns reported here in young adulthood. This does not necessarily mean that they are irrevocable and set in stone for the rest of the individuals' lives. A fundamental issue for practice is whether professional interventions can be designed and effected in ways that make sustained differences to these young people's daily experiences and future prospects.

We have stressed that even the characteristic of shyness, traditionally assumed by many to be an enduring trait, may vary over time and situations; it is, at least in part, an adaptive response to circumstances and experiences (Asendorpf, 2000; Ash et al., 2014; Redmond & Rice, 1998; Wadman et al., 2008). In a similar way, self-esteem can be bolstered by professional and family support (Mann, Hosman, Schaalma, & de Vries, 2004); indeed, while self-esteem was lower in those with LI in the present sample, it was not in the clinical range.

SSE, as a social cognitive construct, is of particular interest for intervention and practice. SSE is likely to be amenable to enhancement in people who have positive experiences to draw upon (Bandura, 1997). There are compelling reasons for paying greater attention to SSE in clinical work with adolescents and young adults with LI. Not only does this measure have much to contribute to client assessment, the strong theoretical background to the concept of SSE provides firm guidelines for intervention strategies (Bandura, 1997; Smith & Betz, 2000). Interventions that theory and evidence (Smith & Betz, 2000) suggest are likely to be of substantial benefit include guiding the young person to set viable self-management goals, modeling successful social strategies, modifying negative expectations of social efficacy and increasing positive expectations, and supporting confidence in the ability to implement new skills.

Our findings also suggest that adolescence may be a sensitive period for the impact of LI on other aspects of personal and social adjustment. Although we do not preclude effects in other age ranges, the finding that language at age 17 years bears more directly on concurrent and subsequent shyness and self-esteem highlights this age range as one to which professionals should be particularly alert to the potential for long-term disadvantage but also for preemptive intervention. In most educational systems, important evaluations and decisions are being made at around this age, and social confidence and educational outcomes are likely to have reciprocal influences.

## Conclusions

This study provides clear evidence that young people with a history of LI are likely to be entering adulthood less socially confident. They are likely to experience lower levels of self-esteem, higher levels of shyness, and, importantly, lower levels of SSE than their peers. This adds to the emerging picture of the developmental continuities and consequences of LI. Future research could investigate the relations among the variables tested here further into adulthood. Some individuals with LI may make progress in their personal and/or occupational lives that will bolster their self-confidence and mitigate their shyness. Others may encounter contexts that are less propitious. Note that the prospect emerges that interventions may be desirable for young adults with LI, and the present findings indicate SSE as a key area of social confidence that calls for practitioners' attention.

The findings also have implications for extending the provision of services. In many parts of the world, these tend to be targeted primarily at childhood, sometimes including adolescence, but rarely beyond, and they often prioritize specific problems (e.g., severity of language difficulties), with less attention paid to the broader impact across the life span (cf. Law et al., 2013). LI can extend into adulthood and is associated with many other aspects of development; services are required that meet both the duration and the breadth of the needs of people with LI.
